# Supplementary material for: Dynamic Evolution of NLR Genes in Dalbergioids
Source: Genes (Basel). 2023 Jan 31;14(2):377. doi: 10.3390/genes14020377 (PMC9956324; doi:10.3390/genes14020377)
Supplement: Supplementary file 1 [file genes-14-00377-s001.zip › supplementary figure/Figure S1.pdf]

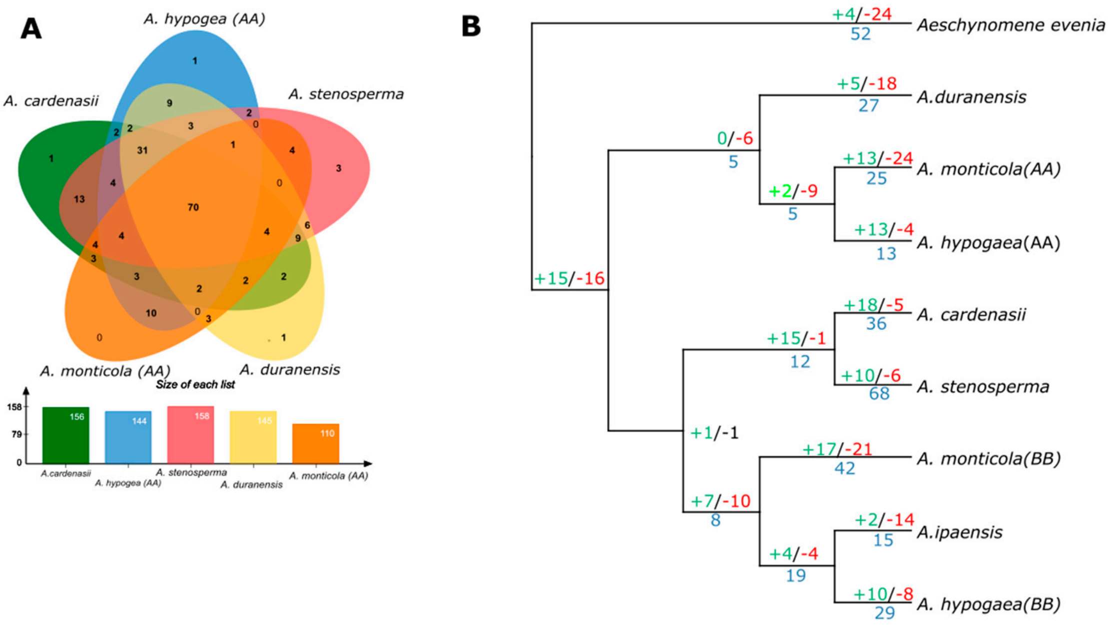

Figure S1. ortholog with gene gain and loss analysis. (A) Represents Venn diagram for five species of *Arachis,* suggesting 70 core components of *Arachis* NLRome. (B) Gene gain and loss tree for *Ae. evenia*, *A. duranensis*, *A. monticola*, *A. hypogaea*, *A. cardenasii*, *A. stenosperma*, and *A. ipaensis*. Green and red color above the node represent gene gain and loss, respectively, with gene duplication numbers shown in blue.
