# Supplementary material for: Dynamic Evolution of NLR Genes in Dalbergioids
Source: Genes (Basel). 2023 Jan 31;14(2):377. doi: 10.3390/genes14020377 (PMC9956324; doi:10.3390/genes14020377)
Supplement: Supplementary file 1 [file genes-14-00377-s001.zip › supplementary figure/Figure S2.pdf]

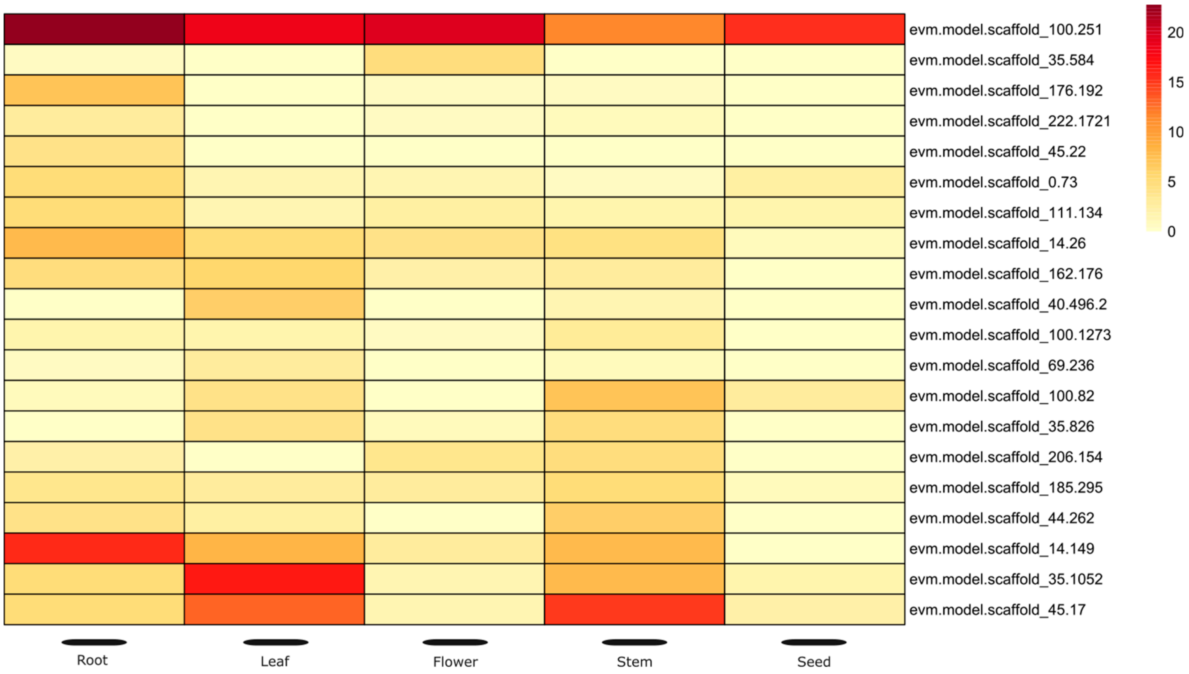

Figure S2. Expression of *NLR* genes in *D. odorifera*. Comparison of *NLR* gene expression in the root, leaf, flower, stem, and seed tissue.
