# Supplementary material for: Dynamic Evolution of NLR Genes in Dalbergioids
Source: Genes (Basel). 2023 Jan 31;14(2):377. doi: 10.3390/genes14020377 (PMC9956324; doi:10.3390/genes14020377)
Supplement: Supplementary file 1 [file genes-14-00377-s001.zip › supplementary figure/Figure S3.pdf]

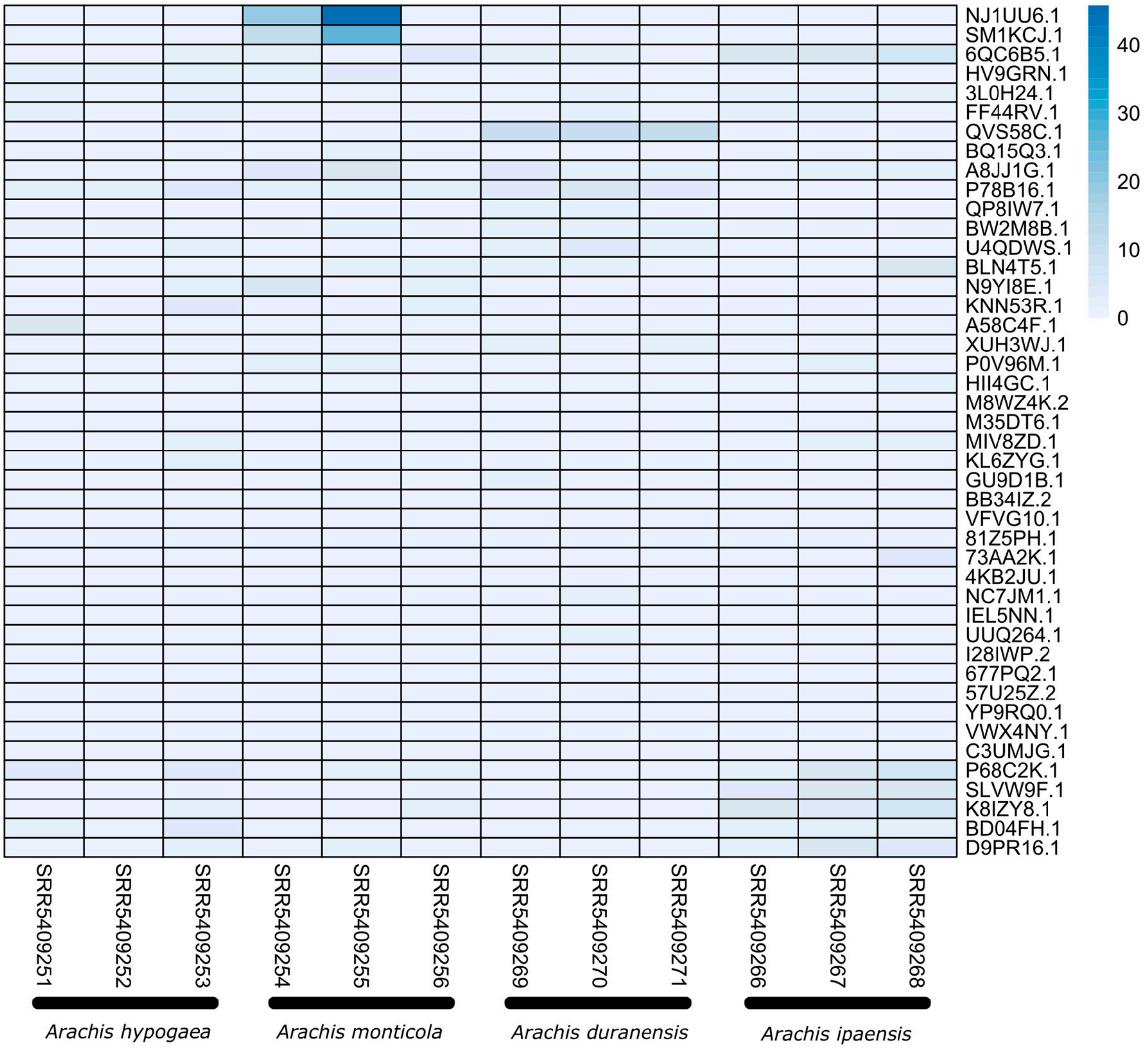

Figure S3. Expression of *NLR* genes in progenitor and tetraploid species. Table S1: The assembled transcriptomes of six *Dalbergioids* species.
